# The Ineligibility of Antipyrin in Pneumonia

**Published:** 1886-09

**Authors:** 


### THE INELIGIBILITY OF ANTIPYRIN IN PNEUMONIA.

Observations of untoward effects produced by newly-introduced drugs, or of their failures in certain affections in which they have been recommended, ought to claim the interest of the practitioner as highly as reports on their medicinal virtues and efficacy. It is a positively objectionable feature of many contributors to the therapeutic press that they explicitly discuss the advantages of drugs, and dwell on the constant expansion of their sphere of usefulness, while paying too little attention to their objectionable aspects, and the instances of their clinical failure. The *Therapeutic Gazette* has repeatedly opened its columns to this unjustly neglected field of therapeutic knowledge. In the case of antipyrin, which is gradually becoming a popular antipyretic, it is of course very desirable to know what the drug cannot do and to which untoward symptoms it can eventually give rise.

Dr. Posadski (vide *Ieshenedjelnaja Klinitscheskaja Gazeta*, No. 30, 1885) reports that he has treated twenty-five cases of croupous pneumonia with antipyrin, giving, daily, doses of 15 grains to 2 drachms, and single doses of 7 to 30 grains. Simultaneously, he treated twenty-three cases of croupous pneumonia with calomel, in order to be enabled to compare the results of both forms of treatment. Calomel was given in doses of 1/8 grain four times daily All patients were vigorous men, ranging between 26 and 30 years of age, and were admitted for treatment at the third or fourth day after the beginning of the affection. The average duration of the pneumonia in the patients treated with antipyrin was 8.1 days; in those treated with calomel, 7.1 days. In the later set of patients, the termination of the affection presented invariably what Posadski calls a critical decline; in the former, a gradual resolution. In other words, the febrile symptoms disappeared completely, and sooner under the calomel treatment, and gradually and later under the antipyrin treatment. In five instances collapse was observed to follow after the use of antipyrin, a condition to which calomel, of course, could never give rise. The effects produced by the two drugs on the pulse and respiration were not materially different In four cases antipyrin caused vomiting, and in two others a peculiar measles-like eruption, of a very itching nature, affecting both skin and mucous membranes. In eleven cases the urine presented a singular dark cherry coloration, and contained a large quantity of antipyrin. The bodily weight showed a greater daily fall under calomel than under antipyrin, though its rise proceeded more quickly under the former drug.

The action of antipyrin on the temperature curves is, according to our observer, neither a constant nor a prompt one, and alongside of the above-mentioned other disadvantages does not justify the exhibition of the drug in pneumonia.—*Therapeutic Gazette.*

